# Supplementary material for: The Human Connectome Project 7 Tesla retinotopy dataset: Description and population receptive field analysis
Source: J Vis. 2018 Dec 28;18(13):23. doi: 10.1167/18.13.23 (PMC6314247; doi:10.1167/18.13.23)
Supplement: Supplement 1 [file jovi-18-13-03_s01.pdf]

Appendix: The HCP 7T Retinotopy Dataset in BALSA and Connectome Workbench

1. Introduction.

The HCP 7T retinotopy dataset is freely available via the BALSA database as downloadable scene files that can be viewed using Connectome Workbench. Each scene file contains multiple individual scenes that display retinotopic and associated data in an arrangement that provides a useful starting point for visualization and further analysis. After downloading, scenes can be displayed in 'wb_view', but **you will need Connectome Workbench v. 1.3 or higher** (https://www.humanconnectome.org/software/get-connectome-workbench), **because the scenes rely on several recently incorporated features.**

The BALSA URL for this study is https://balsa.wustl.edu/study/show/9Zkk
The primary scene file ("Retinotopy_HCP_7T_181_Fit1.scene"; https://balsa.wustl.edu/sceneFile/show/7Nrj) shows retinotopy results for all 181 7T subjects with retinotopy data displayed on various geometries (spheres, flatmaps, etc.) on the "fs_LR" surface mesh aligned using the "MSMAll" areal feature-based alignment. We encourage investigators to use this dataset, which is in the original space in which the retinotopic analysis was carried out and also the original space of the HCP_MMP1.0 multimodal cortical parcellation and a growing number of other datasets. The primary scene file shows the retinotopic analysis fit for the whole dataset (fit1). Additional scene files provide results from (i) analyzing two subgroups of retinotopy subjects (90- and 91-subject groups), (ii) Data from the first half of each run of each subject (fit2) and the second half of each run of each subject (fit3), and (iii) retinotopy results mapped to the fsaverage atlas space.

We briefly describe the layout of the first scene in the primary scene file and give useful navigation tips, as this scene has commonalities with other scenes in this and the other scene files. We then show figure previews for the remaining scenes in the primary scene file, followed by a listing of additional scene files for the 90 and 91 subject datasets and the fit2 and fit3 analyses, plus illustrations of the two scenes in the fsaverage scene file illustrating some of the limitations of mapping the data to fsaverage using nearest neighbor interpolation and displaying it on the fsaverage folding pattern as is done in the main text. The final scene shows data similar to Supplementary Figure 1, comparing the Full vs. ON/OFF task models.

Retinotopy_HCP_7T_181_Fit1.scene: https://balsa.wustl.edu/sceneFile/show/7Nrj

2. Viewing and navigating the first retinotopy scene.

Figure A1 shows the group average (n = 181) maps of all of the retinotopic data (fit1) polar angle, eccentricity, pRF size, and myelin viewed on spherical maps of the left and right hemisphere, oriented for maximal coverage of retinotopic areas (slightly different than the orientation used for the main text figures). The HCP_MMP1.0 borders appear as an overlay (white contours); the underlay is the group average folding map (FreeSurfer's curvature measure) from the same 181 subjects aligned with the same MSMAll registration algorithm; this underlay is visible only in transparent regions where the model R^2 is less than 9.8%. Using the average folding map derived from the same subjects used for the retinotopy analysis provides a more realistic representation of folding-function relationships in this

dataset than if one uses the fsaverage map of average folding from a separate set of subjects aligned using a different registration algorithm (see Figure A10 for a direct comparison).

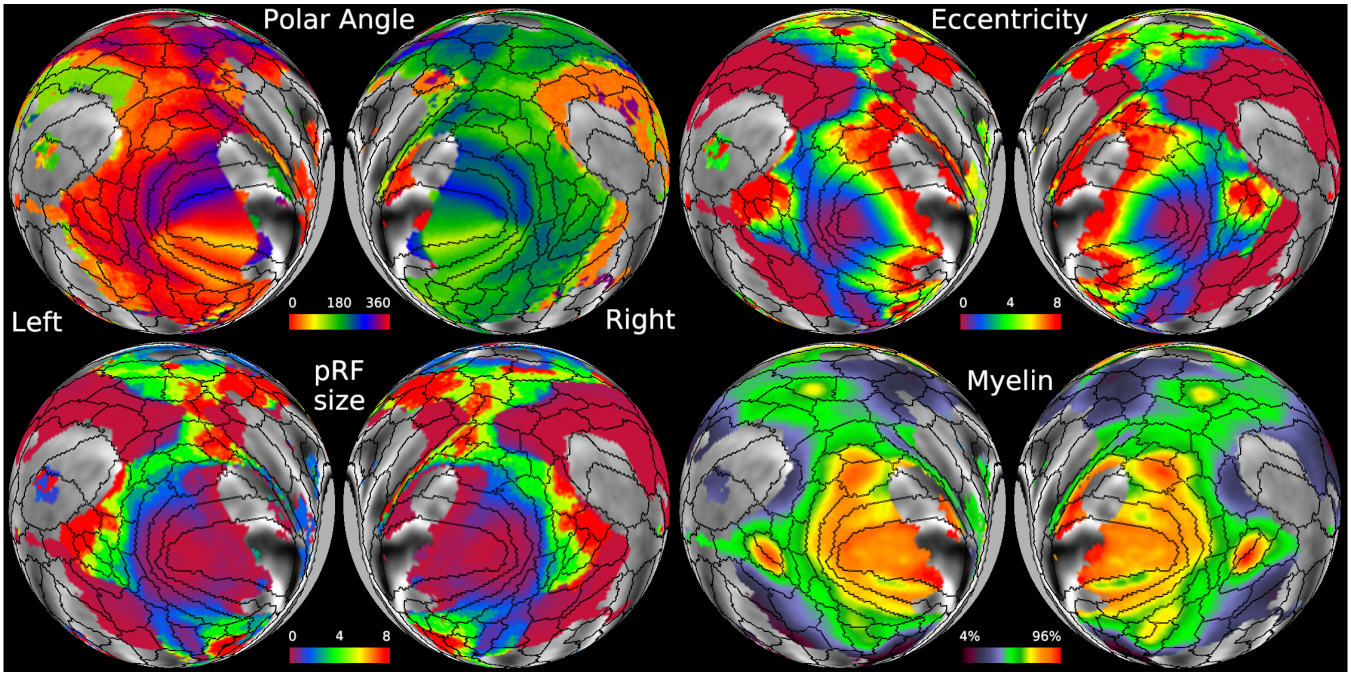

Figure A1. Group average polar angle, eccentricity, pRF size, and myelin maps viewed on spherical maps with the light shading omitted in order to make the view look flatter. For the polar angle map (0 to 360), 0 (and 360) degrees corresponds to the horizontal meridian in the right visual hemifield (left hemisphere), whereas 180 degrees corresponds to the horizontal meridian in the left visual hemifield. Thresholding of all maps is at 9.8% of the variance explained for the group in this and other group average figures, showing the group average folding map underneath the thresholded portions. Data at https://balsa.wustl.edu/k0Z2.

Switching from Tile Tabs to single tab views. This scene opens in "Tile Tabs" mode, showing four tabs concurrently. To view in single-tab mode, select the tab of interest then select View: Exit Tile Tabs or use a keystroke shortcut – CMD-M (Mac) or CTRL-M (Linux). To return to Tile Tabs, select View: Enter Tile Tabs, repeat the CMD-M/CTRL-M shortcut, or press "Show" in the Scenes dialog window.

View other maps using the Overlay Toolbox. Each scene includes many loaded but initially invisible files that can be displayed using the Overlay Toolbox. For example, instead of the default polar angle "0 – 360" palette (0 = red = right hemifield horizontal meridian, with distinct hues for the left and right visual hemifield), a mirror-symmetric palette (analogous to Fig. 4 of the main text) can be viewed by toggling off the top polar angle layer in the Overlay Toolbox menu. Toggling off the third layer ("PolarAngleMirror") reveals the complete average curvature map in layer 4. For each layer, the File pulldown menu enables selection of any of the currently loaded maps (mostly in 'dscalar' format).

Comparisons with the Wang2015 retinotopic parcellation. The Features Toolbox (on the right, open by default for this scene) enables turning off of the HCP_MMP1.0 areal borders (uncheck the two checked boxes labeled "Q1-Q6_RelatedParcellation210.R…" and "Q1-Q6_RelatedParcellation210.L…") and turning on the Wang2015 retinotopic borders ("wang2015.L….." and "wang2015.R…").

3. Additional scenes in the primary scene file.

Figures A2 – A4 show retinotopy data on flatmaps, inflated surfaces, and average midthickness surfaces in the same layout as for Figure A1.

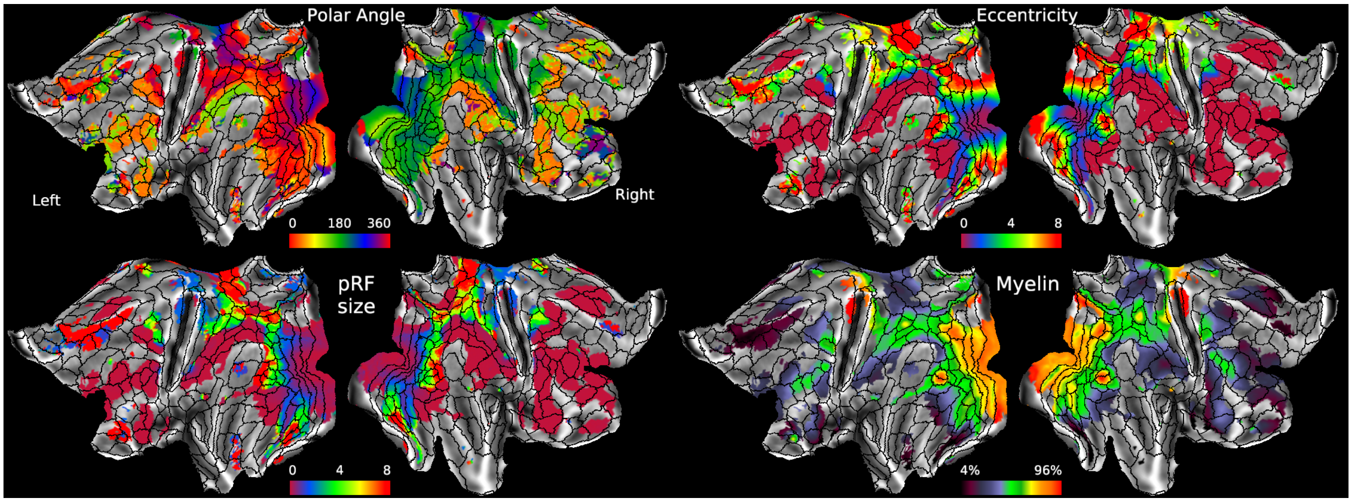

Figure A2. Group average polar angle, eccentricity, pRF size, and myelin maps viewed on flat maps of the left and right hemispheres. Data at https://balsa.wustl.edu/0gVl.

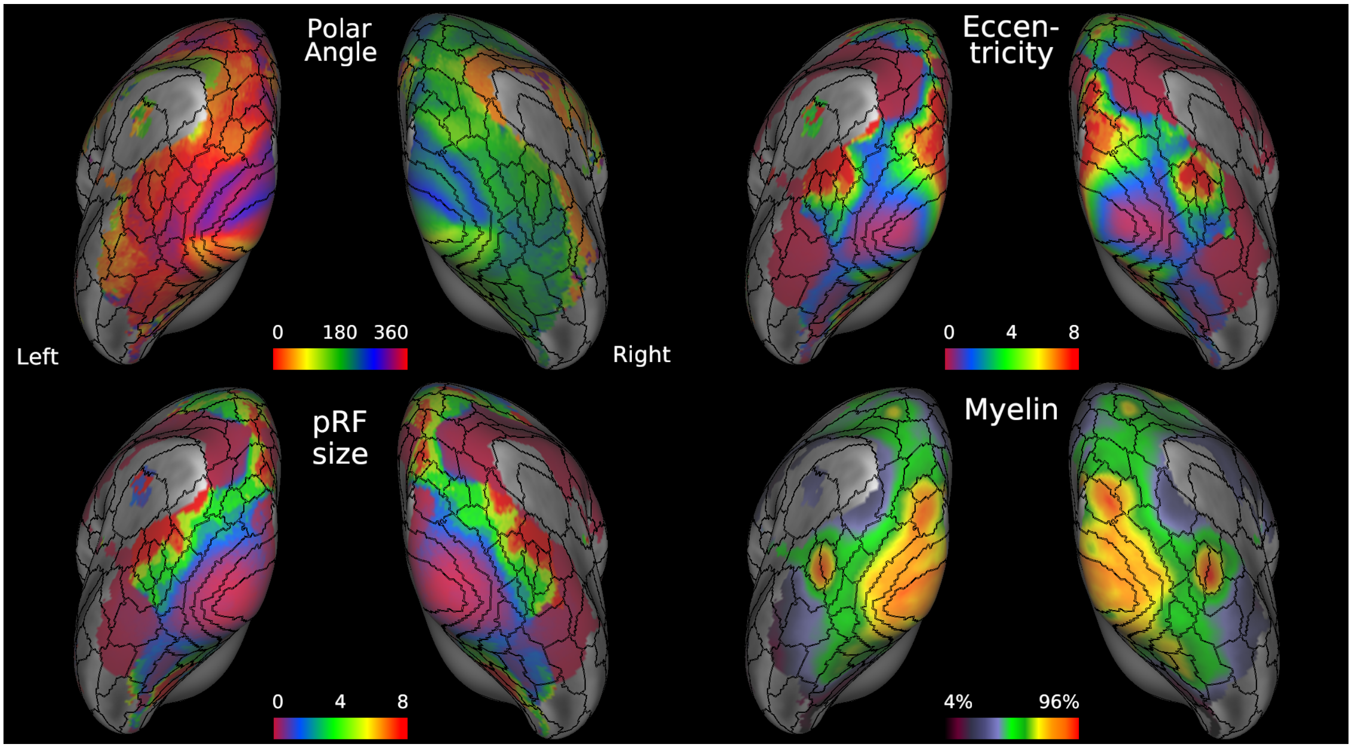

Figure A3. Group average polar angle, eccentricity, pRF size, and myelin maps viewed on inflated left and right hemispheres. Data at https://balsa.wustl.edu/2BzG.

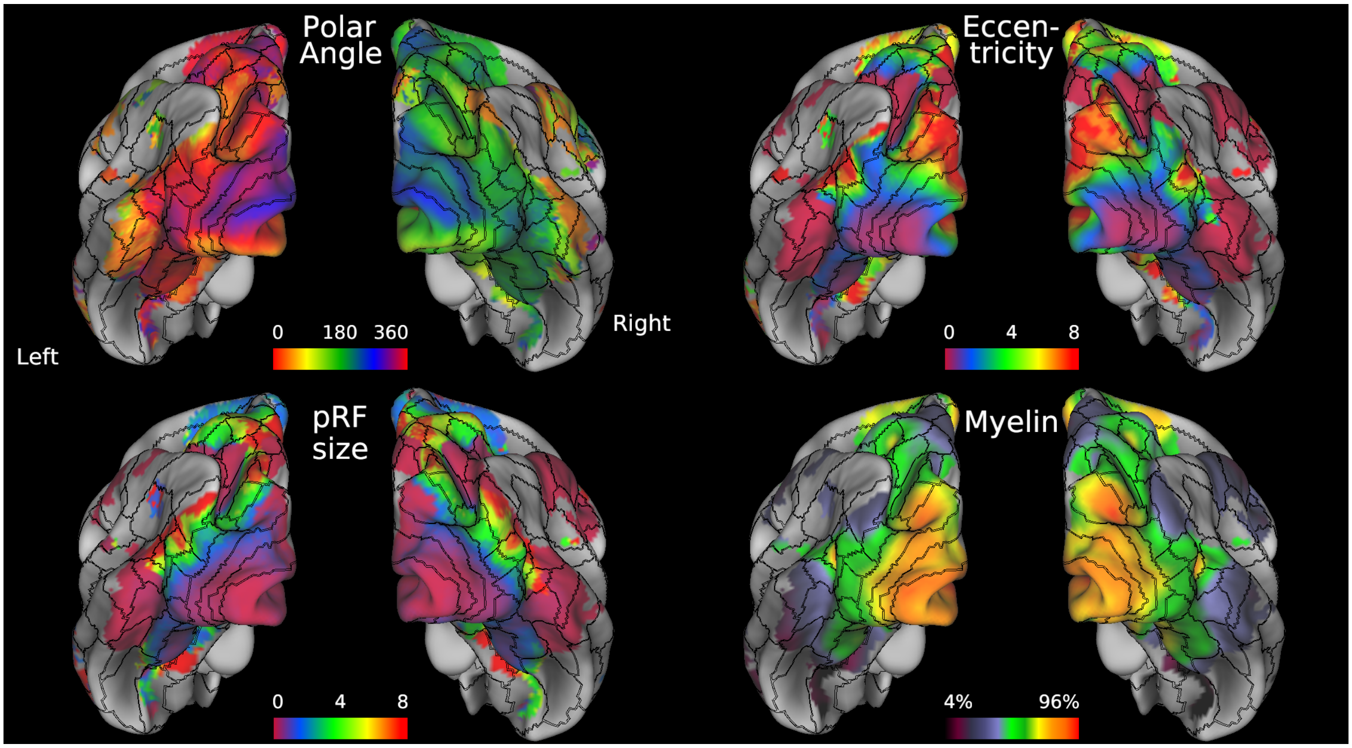

Figure A4. Group average polar angle, eccentricity, pRF size, and myelin maps viewed on average left and right midthickness surfaces. Data at https://balsa.wustl.edu/r15G.

Subcortical retinotopy maps. Figure A5 shows group average retinotopy maps on subcortical volume slices.

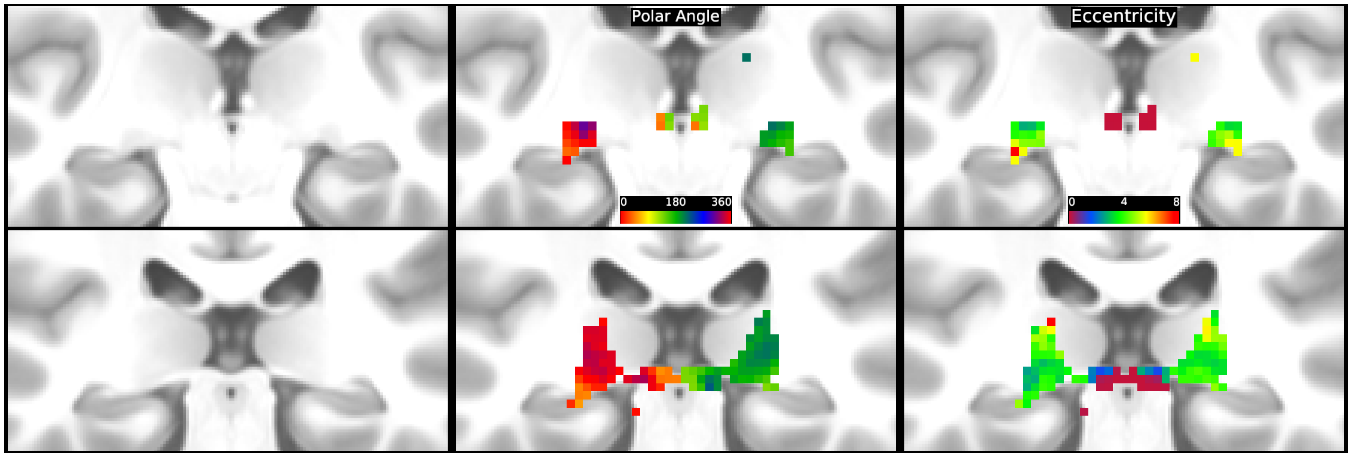

Figure A5. Group average polar angle (middle) and eccentricity (right) overlaid on average T1w coronal slices (left) at the level of the LGN (top row, y = -25) and at the level of the superior colliculus and pulvinar (bottom row, y = -30), as in Fig. 6 of main text. Data at https://balsa.wustl.edu/xlq2.

Individual-subject maps. Figure A6 shows retinotopic and myelin maps for HCP subject 100610 (lowest subject number of the 181 studied), displayed on spherical maps in the same arrangement as Figure A1 and akin to Figure 7 of the main text. Retinotopy for each individual can be examined and compared using the scroll bar to the left of the Map layer for PolarAngle; since the maps are yoked in this scene, changing one map changes those in the other tabs as well.

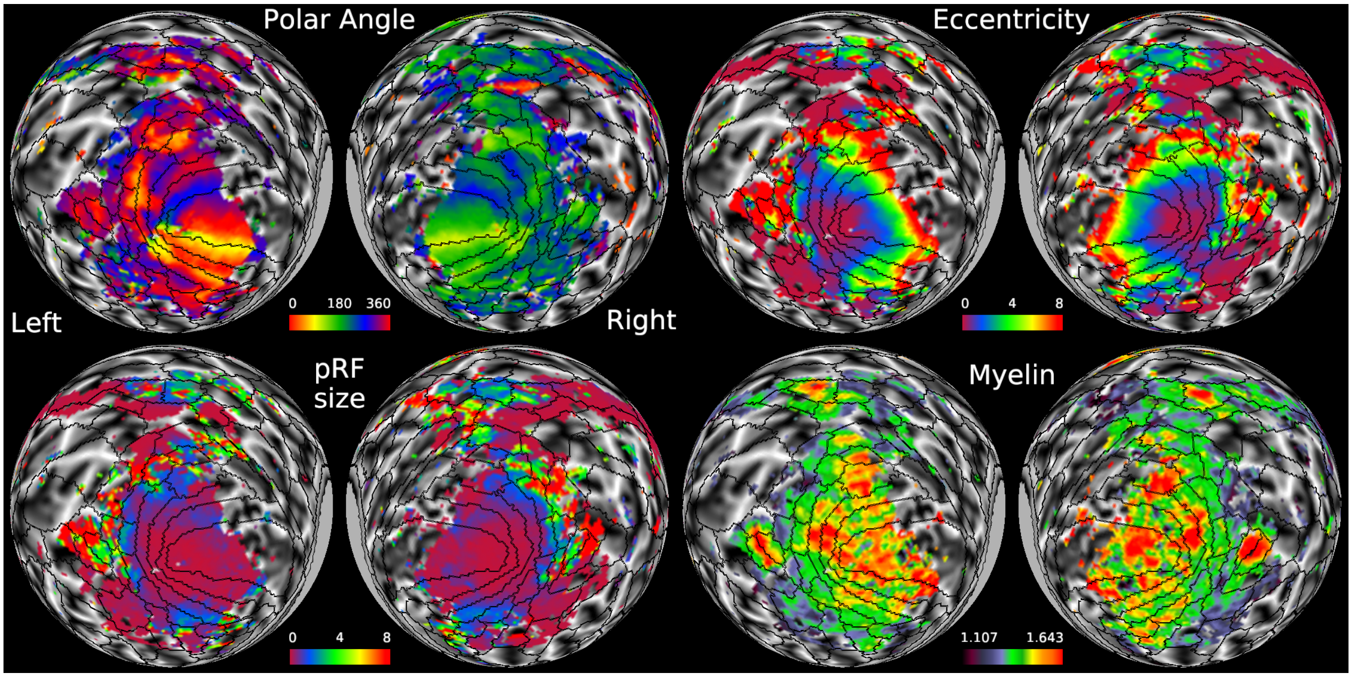

Figure A6. Individual-subject polar angle, eccentricity, pRF size, and myelin maps viewed on top of individual subject folding maps for the left and right spherical surfaces. Thresholding for all individual subjects is at 2.2% of the variance explained for that subject. https://balsa.wustl.edu/PL72.

A nonexhaustive list of subjects with reasonable signal quality but potentially atypical polar angle retinotopy (organization not just alignment to average) include: map 5 (subject 105923, blue = inf VM), 7 (109123, ditto), 9 (111514, ditto), 15 (125525, upper and lower), 41, 43 (LH – foveal green ipsi HM), 81, 86, 91, 105, 174.

An alternate arrangement for viewing retinotopy. Figure A7 shows group average retinotopy on spherical maps with right hemispheres all in the top row and left hemispheres in the bottom row. This can be convenient when inspecting data in the Tile Tabs mode, but it has a less convenient aspect ratio upon switching to single tab mode.

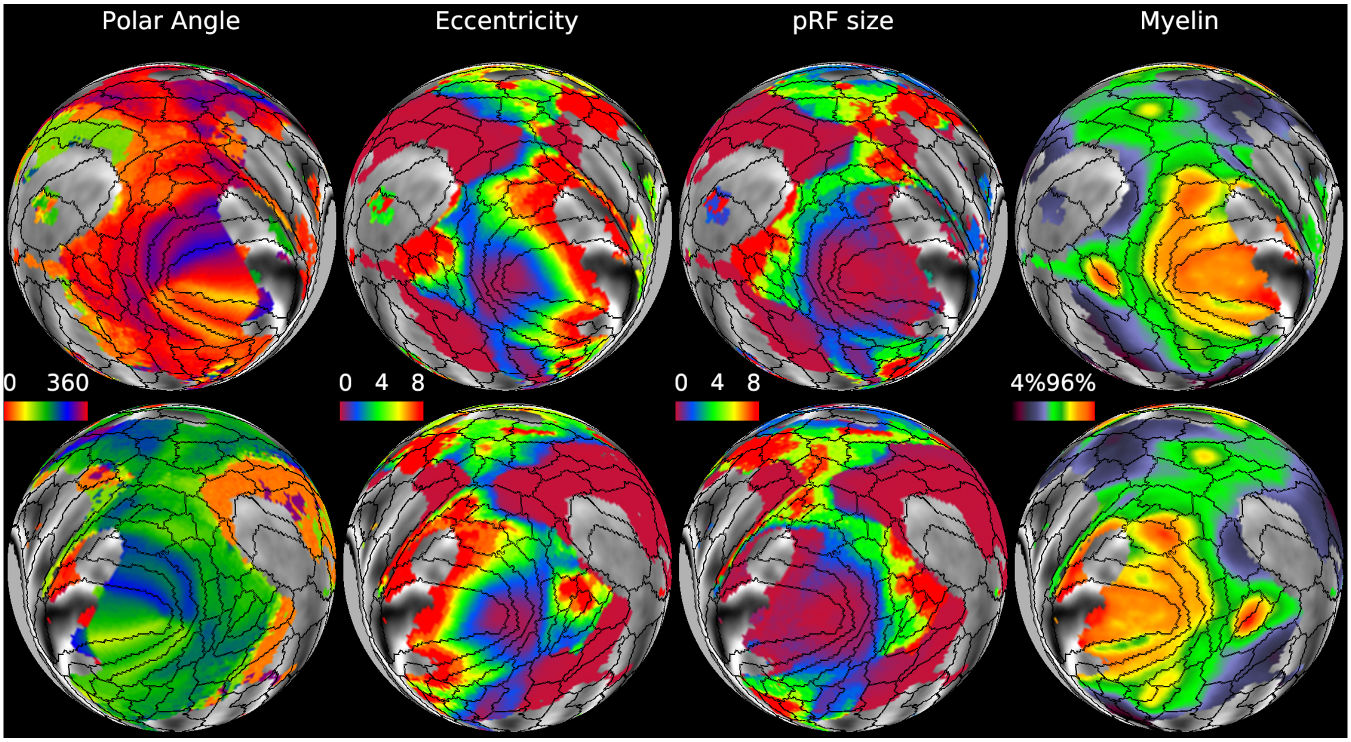

Figure A7. Group average polar angle, eccentricity, pRF size, and myelin maps viewed on average left and right spheres in a left on top; right on bottom configuration. Data at https://balsa.wustl.edu/7ZMj.

Comparing HCP_MMP1.0 and Wang et al. (2015) parcellations. Figure A8 shows the Wang et al. (2015) retinotopic parcellation (colored parcels) compared to the Glasser et al. (2016) HCP_MMP1.0 parcellation (black borders). There is good agreement for early visual areas (V1 – V3 and V3A) bilaterally, aside from the retinotopic parcel extent reflecting incomplete mapping of the visual field. For other areas the alignment is modest to poor and likely results from differences in alignment method and differences in parcellation (e.g. around V4, see Glasser et al 2016a).

Retinotopy_HCP_7T_Atlas.scene: https://balsa.wustl.edu/sceneFile/show/vrVl

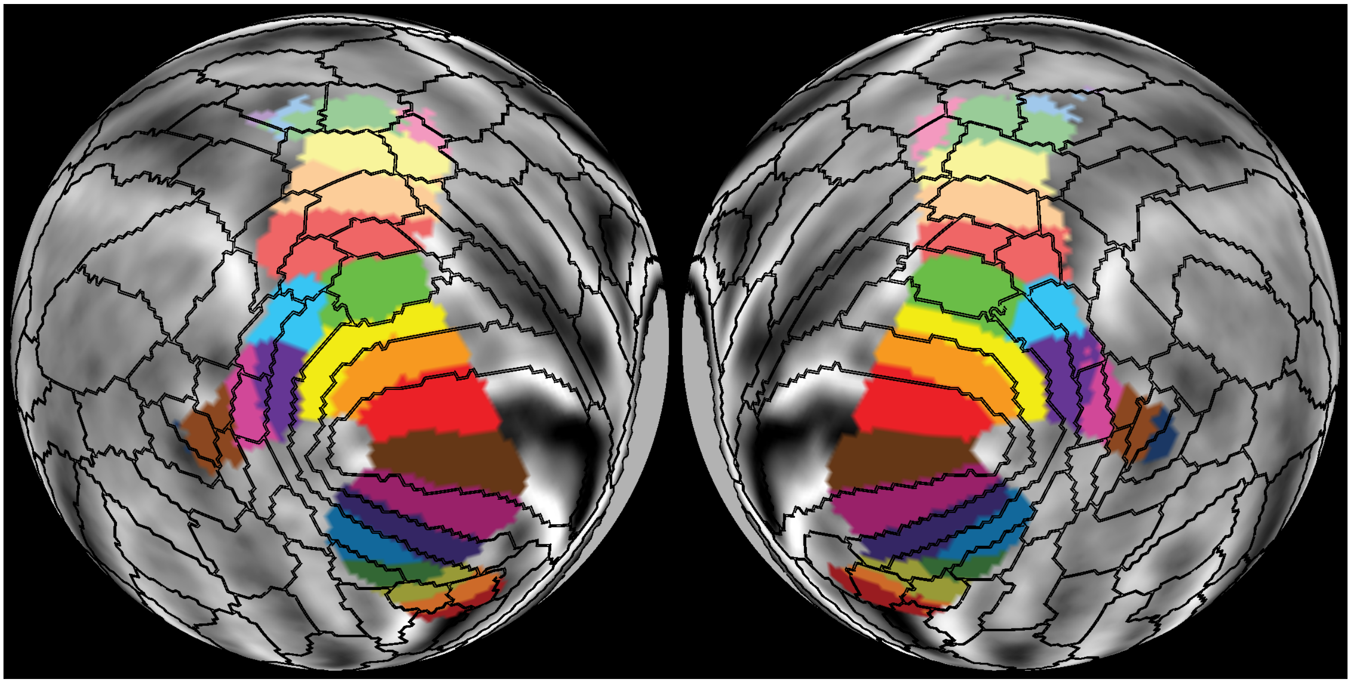

Figure A8. Wang et al. (2015) retinotopic parcellation (colored parcels) compared to Glasser et al. (2016) HCP_MMP1.0 parcellation (black borders). Data at https://balsa.wustl.edu/xlxx.

4. Fit2 and Fit3 retinotopic data.

The scene files for the fit2 (first half of each run of each subject) and fit3 (second half of each run of each subject) retinotopic analyses (see Methods in main text) replicate the scenes shown above (Figures A1 – A7). Here we provide BALSA URLs for the corresponding scene files; linking to these provides access to the individual scene URLs as well.

Retinotopy_HCP_7T_181_Fit2.scene: https://balsa.wustl.edu/sceneFile/show/LjNP

Retinotopy_HCP_7T_181_Fit3.scene: https://balsa.wustl.edu/sceneFile/show/3jG6

5. 90-subject and 91-subject retinotopic data.

The scene files for the 90-subject and 91-subject subgroups of retinotopic analyses (see Methods in main text) replicate the scenes shown above (Figures A1 – A5 and A7). Here we provide BALSA URLs for the corresponding scene files; linking to these provides access to the individual scene URLs as well.

Retinotopy_HCP_7T_90_Fit1.scene: https://balsa.wustl.edu/sceneFile/show/7Nkj

Retinotopy_HCP_7T_91_Fit1.scene: https://balsa.wustl.edu/sceneFile/show/lp0z

6. Retinotopy and folding data mapped to fsaverage atlas.

Figures A9 and A10 show data mapped from the fs_LR 32k mesh to the hemisphere-specific fsaverage (fs_L and fs_R) atlas surfaces. They are in scene file:

Retinotopy_HCP_7T_181_fsaverage.scene: https://balsa.wustl.edu/sceneFile/show/jGw9

Mean curvature for fsaverage vs MSMAll. Figure A9 shows maps of mean curvature (folding) computed from the fsaverage atlas (column 1) and the average across the 181 7T retinotopy subjects aligned using MSMAll (column 2). Columns 3 and 4 show the same maps binarized for positive (gyral) vs negative (sulcal) average folding values. The lower values of mean curvature for the MSMAll reflect its greater reliance on using areal features for alignment, in contrast to fsaverage reliance on alignment based only on folding even in regions of high folding variability. Additionally, the fsaverage curvature map appears to have been recalculated on the fsaverage average surface itself, rather than being the average of the curvature maps across the subjects that generated fsaverage. Data are displayed on the fsaverage ("fs_L" and "fs_R") surface meshes. Although some features are grossly similar (e.g. calcarine sulcus), many are quite different between the two folding maps. Thus, it can be misleading to interpret the relationships between retinotopic data and folds of different datasets aligned using different registration algorithms or folding maps computed from an average surface vs the average map across subjects.

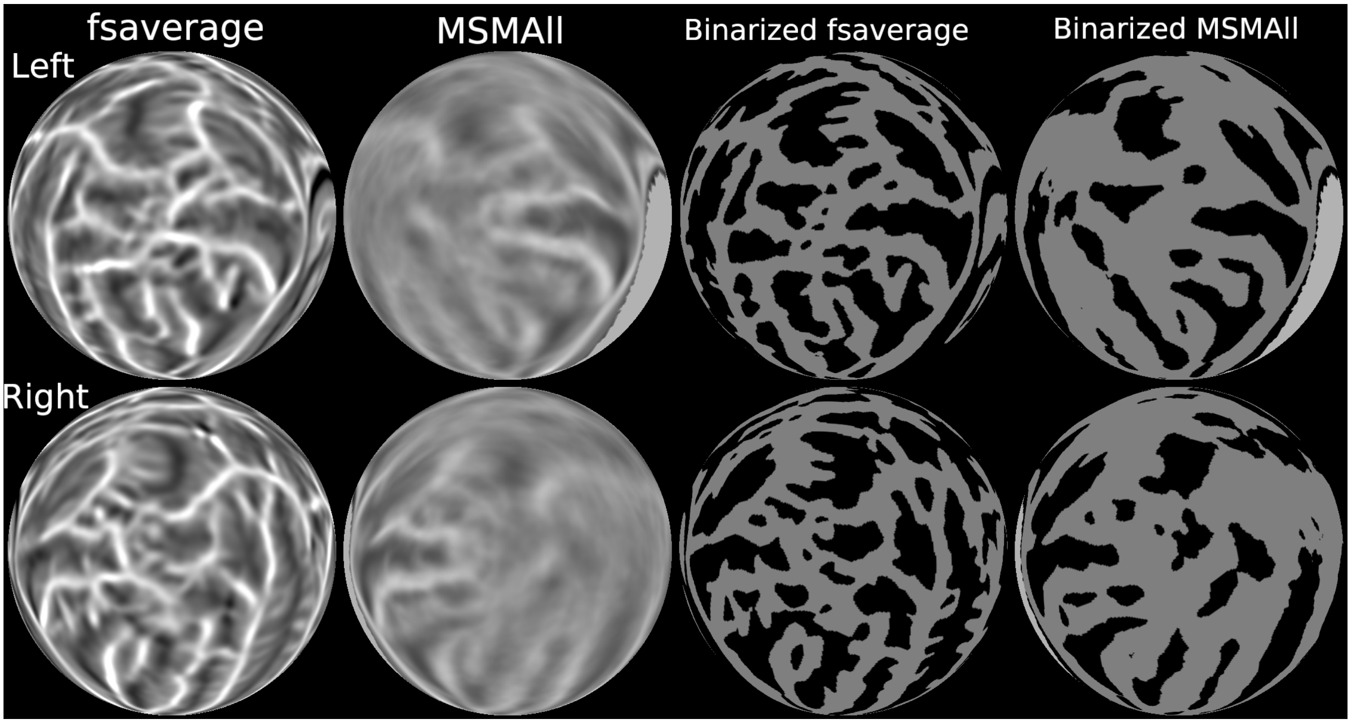

Figure A9. Maps of mean curvature (folding) computed from fsaverage (column 1) and from the 181 7T retinotopy subjects aligned using MSMAll (column 2). Columns 3 and 4 show the same maps binarized for positive (gyral) vs negative (sulcal) average folding values. Data are displayed on the fsaverage ("fs_L" and "fs_R") surface meshes. Top row: left hemisphere spheres. Bottom row: right hemisphere spheres. Data at https://balsa.wustl.edu/VN4g

Figure A10 shows the retinotopy data from the fs_LR 32k mesh to the hemisphere-specific fsaverage (fs_L and fs_R) atlas surfaces using two methods. One was the nearest neighbor method as described in the main Methods, which suffers from "pixelation" when upsampled from the fs_LR 32k mesh on which the data were computed to the 164k fs_L and fs_R fsaverage meshes. The other was an adaptive barycentric method, which is inherently smoother. However, for the polar angle and eccentricity maps, we first converted to Euclidean coordinates, in order to avoid discontinuities arising

solely from representation, followed by adaptive barycentric area resampling, then conversion back into polar angle and eccentricity.

Note that the fsaverage datasets are much larger (and slower to load) because they were upsampled to the 164k FreeSurfer mesh, rather than the fs_LR 32k mesh in which the retinotopy analyses were carried out.

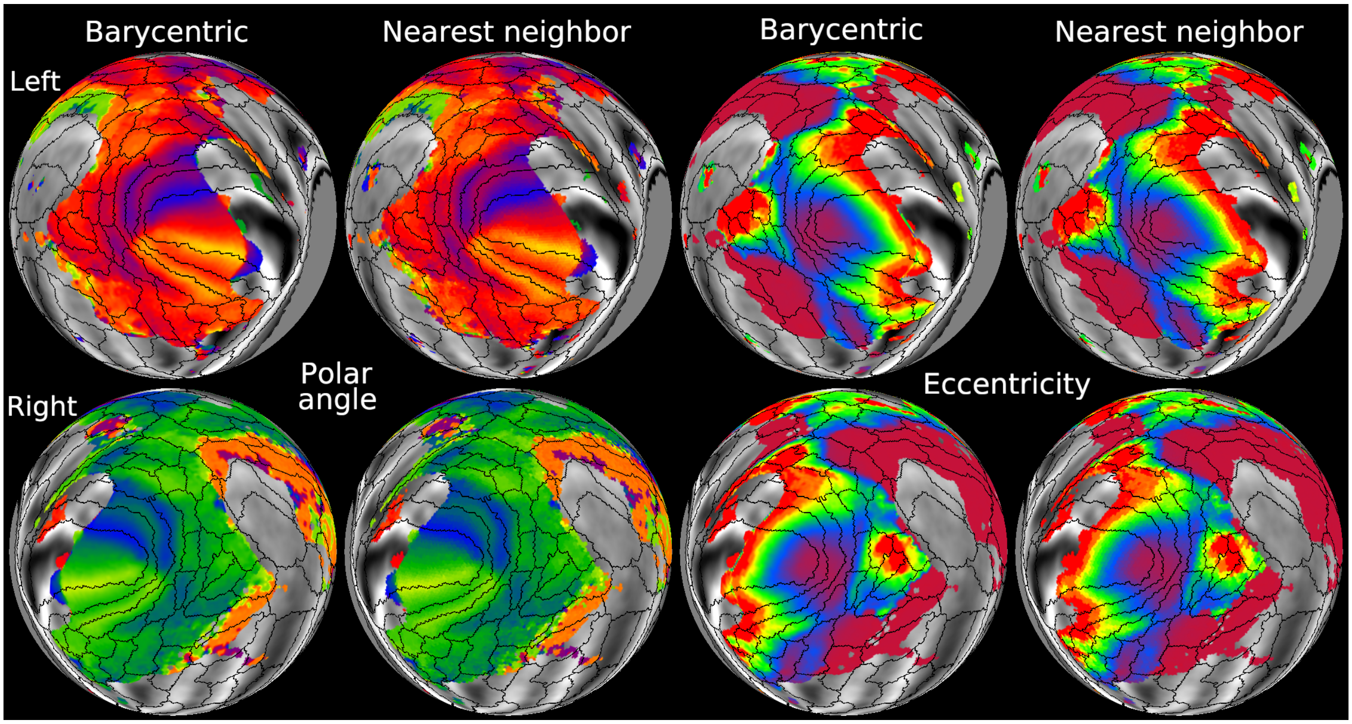

Figure A10. Group average polar angle (columns 1 and 2) and eccentricity (columns 3 and 4) maps viewed on fsaverage spheres. Thresholding is at 9.8% of the variance explained. Columns 1 and 3: Barycentric mapping from HCP fs_LR to fsaverage mesh. Columns 2 and 4: Nearest neighbor mapping from HCP fs_LR to fsaverage mesh. HCP_MMP1.0 areal borders resampled to fsaverage are displayed in all panels. Data at https://balsa.wustl.edu/3zNv

7. Full versus ON/OFF Task Models.

Retinotopy_HCP_7T_181_Fit1_R2.scene:

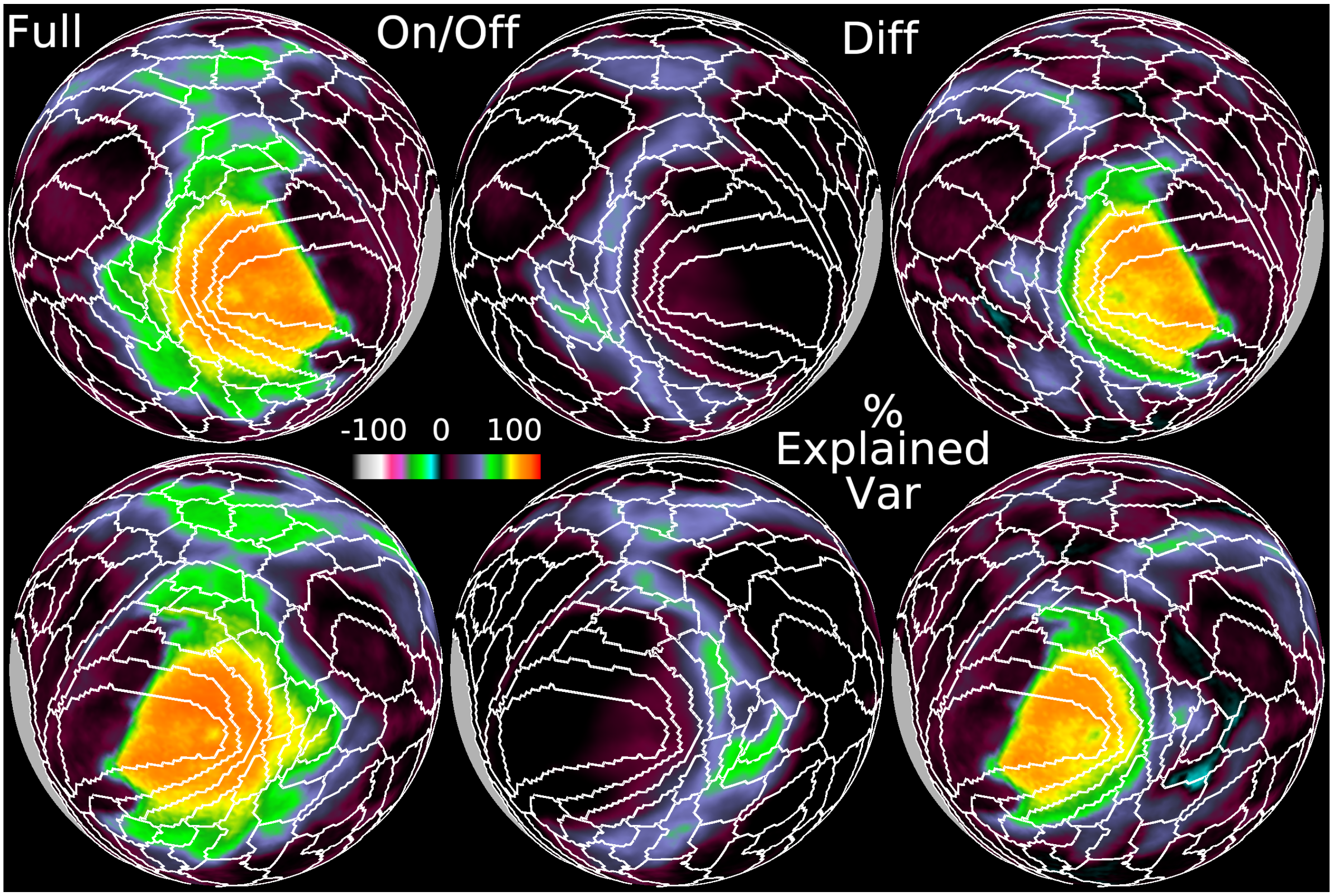

Figure A11. Comparison of full vs. ON/OFF task models for the 32k fs_LR mesh for the group average 181 subject dataset. The left most panel shows the % variance explained for the full retinotopic task model. The center panel shows the % variance explained for the stimulus ON/OFF task model. The right most panel shows the difference between the full model and the stimulus ON/OFF model, i.e. the % variance explained that is related to spatially selective signals. Data at https://balsa.wustl.edu/gj8D
