# Supplementary material for: The Human Connectome Project 7 Tesla retinotopy dataset: Description and population receptive field analysis
Source: J Vis. 2018 Dec 28;18(13):23. doi: 10.1167/18.13.23 (PMC6314247; doi:10.1167/18.13.23)
Supplement: Supplement 2 [file jovi-18-13-03_s02.pdf]

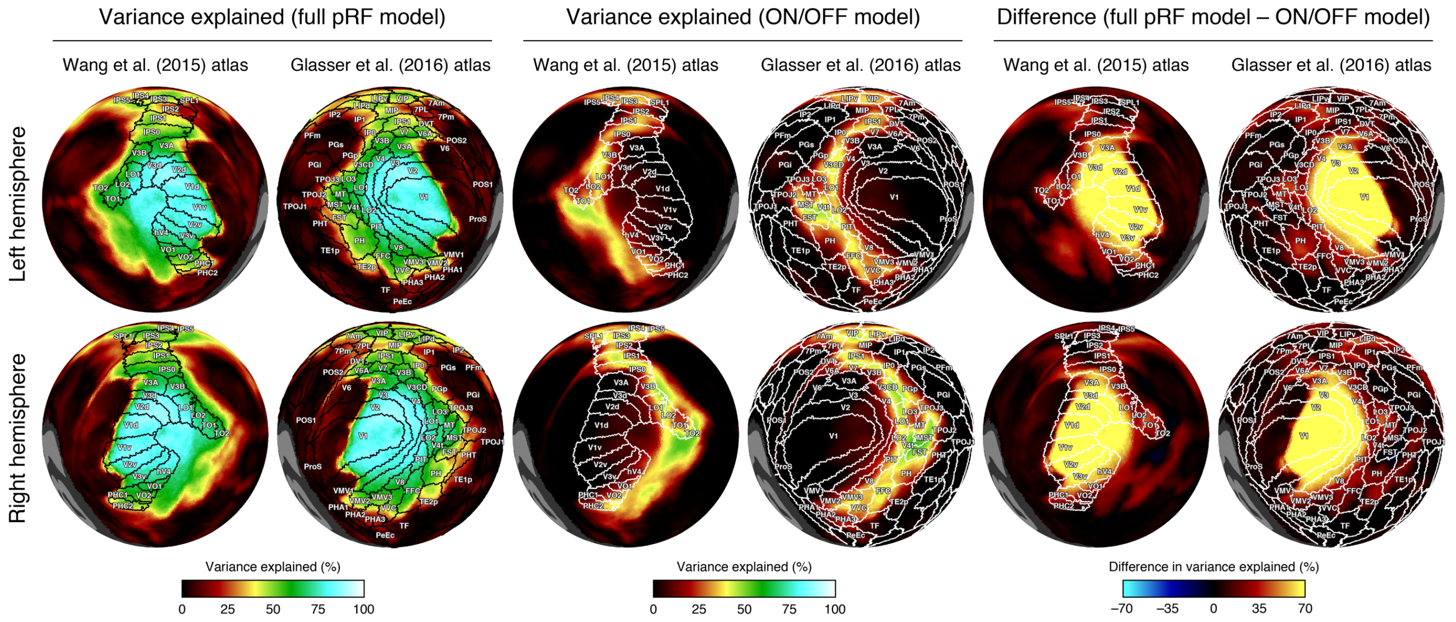

Supplementary Figure 1. Comparison of full pRF model to a simple ON/OFF model. It is possible that the pRF model, when fitted to a grayordinate, successfully explains variance in the time-series data, even if the grayordinate possesses little or no spatial selectivity. To assess this issue, we constructed a simple model that is sensitive only to the presence or absence of the retinotopic mapping stimulus. In this model (code available on the OSF web site), the stimulus-related time-series is computed as the convolution of the canonical HRF with a time-series whose value is 1 when a stimulus is present in the visual field and 0 when a stimulus is absent. This ON/OFF model is an asymptotic case of the pRF model as pRF size approaches infinity. This figure depicts for the group average (S184), the variance explained by the full pRF model, the variance explained by the ON/OFF model, and the difference between the two. As expected, the ON/OFF model fares poorly in early visual areas (V1–V3) but performs increasingly well in later visual areas. This is consistent with the fact that later visual areas have increasingly large pRFs. However, even in lateral (e.g. LO-1/2, TO-1/2), ventral (e.g. hV4, VO-1/2), and parietal regions (e.g. MIP, IP0, IP1), the full pRF model explains substantially more variance than the ON/OFF model. This indicates that these regions are not only visually responsive but also exhibit some degree of spatial selectivity.
