# Supplementary material for: The Human Connectome Project 7 Tesla retinotopy dataset: Description and population receptive field analysis
Source: J Vis. 2018 Dec 28;18(13):23. doi: 10.1167/18.13.23 (PMC6314247; doi:10.1167/18.13.23)
Supplement: Supplement 3 [file jovi-18-13-03_s03.pdf]

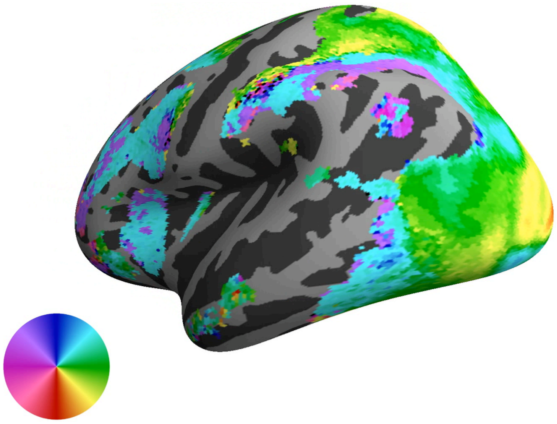

Supplementary Movies 1–12. pRF results on dynamic rotating cortical surfaces. Each movie shows group-average pRF model solutions (S184) on the inflated *fsaverage* surface. The format is the same as in Figure 4. There are a total of 2 hemispheres × 6 maps (curvature, angle, eccentricity, zoomed eccentricity, pRF size, variance explained) = 12 movies. The movies are accessible at the OSF web site (https://osf.io/bw9ec/).
